# Determinants and Factors Associated with the Maintenance of Exclusive Breastfeeding after Hospital Discharge after Birth

**DOI:** 10.3390/healthcare10040733

**Published:** 2022-04-14

**Authors:** Sergio Martínez-Vázquez, Antonio Hernández-Martínez, Julián Rodríguez-Almagro, Rocío Adriana Peinado-Molina, Juan Miguel Martínez-Galiano

**Affiliations:** 1 Department of Nursing, University of Jaen, 23071 Jaen, Spain; svazquez@ujaen.es; 2 Department of Nursing, Faculty of Nursing of Ciudad Real, University of Castilla-La Mancha, 13071 Ciudad Real, Spain; antonio.hmartinez@uclm.es (A.H.-M.); julianj.rodriguez@uclm.es (J.R.-A.); 3 Hospital Complex of Jaen, 23007 Jaen, Spain; rpmolina@ujaen.es; 4 Consortium for Biomedical Research in Epidemiology and Public Health (CIBERESP), 28029 Madrid, Spain

**Keywords:** exclusive breastfeeding, associated factors, breastfeeding, obstetric interventions, obstetric outcomes, infant feeding

## Abstract

The benefits of exclusive breastfeeding are well known for both mother and baby. Despite this, rates of exclusive breastfeeding remain low. The present study aimed to determine the factors associated with the maintenance of this type of feeding after being discharged from the hospital after childbirth. A cross-sectional study was carried out with 1200 postpartum women in Spain. Sociodemographic, obstetric, and neonatal data were collected. Odds ratios (OR) and adjusted odds ratios (aOR) with 95% confidence intervals were calculated. Early breastfeeding initiation was identified as a factor that favors breastfeeding after hospital discharge (aOR: 2.47; 95%CI: 1.77, 3.45). Other factors that favor breastfeeding after discharge included the woman feeling very supported by her partner during pregnancy, childbirth, and the puerperium (aOR: 2.54; 95%CI:1.30, 5.00) and having previously breastfed other children (aOR: 1.97; 95%CI: 1.40, 2.76). Among the factors that hindered exclusive breastfeeding after discharge were multiple or twin pregnancies (aOR: 0.31; 95%CI 0.12, 0.83), induction of labor (aOR: 0.73; 95%CI: 0.53, 0.99), admission of the newborn to the neonatal intensive care unit (NICU) (aOR: 0.31; 95%CI 0.19, 0.52), using epidural pain relief during labor (aOR: 0.41; 95%CI: 0.27, 0.64), or a preterm newborn (aOR: 0.38; 95%CI: 0.21, 0.69). For all these reasons, it is essential to promote certain practices such as the early start of breastfeeding or the induced onset of labor, among others, in order to promote the maintenance of exclusive breastfeeding beyond hospital discharge after childbirth.

## 1. Introduction

Breastfeeding is associated with health benefits for both the newborn and the mother [1]. The World Health Organization (WHO) and the United Nations Children’s Fund (UNICEF) recommend breastfeeding as the exclusive method of early feeding for all babies during the first six months of their lives and advise continuing to breastfeed until two years of age and beyond [2,3]. Breast milk is unique in its nutritional composition; it has immunological and anti-inflammatory properties due to its dynamic bioactivities, which adapt according to the infant’s needs [4]. Therefore, it favors the correct development and growth of the baby [5]. Commercial formulas lack these bioactive compounds, and such compounds are diminished in donated milk [6].

Exclusive breastfeeding (EBF) is associated with multiple benefits. For the mother, it is related to a lower risk of breast and ovarian cancer, a lower risk of type 2 diabetes, [7,8] and facilitates the mother–child bond. For the baby, EBF favors greater neurodevelopment and psychomotor development [5,9,10]. In addition, EBF contributes to reducing prevalent infections in childhood and other diseases such as asthma, dermatitis, childhood obesity, and diabetes. EBF is also associated with a lower risk of necrotizing enterocolitis and sudden infant death [11]. EBF also has a positive psychological impact on mothers, reducing the risk of anxiety and depression. This effect is also replicated in the baby’s development, reducing the risk of anxiety or hyperactivity disorders [12]. 

Success in initiating EBF and its continuation is multifactorial and requires support for the simultaneous implementation of support and different measures at different levels: breastfeeding support policies, educational interventions, breastfeeding promotion, work–family conciliation, or ease of employment [13]. In 1989, the WHO and UNICEF supported the initiation and continuation of breastfeeding with the strategy “Ten Steps for Successful Breastfeeding” within the baby-friendly hospital initiative [14]. In 1981, the World Health Assembly adopted the International Code of Marketing of Breast-milk Substitutes, intended to protect breastfeeding from unethical business practices. Later, in 1991, the Initiative for the Humanization of Birth and Breastfeeding Assistance (Iniciativa Hospital Amigo de los Niños, IHAN, in Spanish and BFHI in English)) was launched to encourage health centers, particularly maternity wards, to adopt practices that protect, promote, and support EBF from birth [15]. 

Despite EBF being the type of infant feeding that provides the greatest benefits to the mother–infant dyad, its overall rates remain low. According to the WHO, between 2006 and 2012, 25% of infants in Europe were breastfed during the first six months—one of the lowest rates in the world [16]. In Spain, EBF rates during the first postpartum weeks have been reported to be around 39% [17], not reaching the minimum target of 50% set by the WHO [18,19]. In 2016, above-average rates were documented in South Asia and Africa, with 43% of children receiving EBF [18,19].

Different factors influence the initiation of EBF, such as skin-to-skin contact, maternal education level, the type of professional who attends the birth as well as their behavior, whether the woman had access to an epidural, whether the birth was induced, admission of the baby to intensive care, or premature birth [18,20,21,22,23,24,25,26,27,28]. Knowing the impact of all of these on the initiation and subsequent maintenance of EBF is fundamental to be able to support and implement policies that favor EBF.

Despite the worldwide policies promoted by different administrations and organizations such as the WHO or UNICEF, along with all the known benefits of EBF and the known determinants associated with the establishment and maintenance of EBF, the rates of EBF are far from those that are recommended. For this reason, it remains necessary to verify the known factors that influence the maintenance of EBF after hospital discharge, as well as to identify other, possible new factors that influence its maintenance beyond hospital discharge after childbirth. Therefore, this study aimed to determine the factors associated with the maintenance of EBF after hospital discharge.

## 2. Materials and Methods

### 2.1. Design and Subject Selection 

An analytical observational study was carried out on women who gave birth in Spain during 2019. The inclusion criteria were established as women aged 18 years or older at the beginning of the study and who wanted to breastfeed their babies. Those women who had difficulties communicating due to cognitive problems, sensory disabilities, or language barriers were excluded.

The sample size was estimated following the maximum modeling model for multivariate analysis, which requires a minimum of 10 events for each independent variable included in the model. Thus, considering a minimum of 20 initial independent variables, a minimum of 200 women exclusively breastfeeding after discharge would be needed. Taking into account the prevalence of exclusive breastfeeding of 47% (the intermediate point between the prevalence limits observed in the literature, ranging from 37% to 57%), [13,29,30] a minimum of 425 women would be needed. Given the possible loss of participants during the study and to improve statistical power, it was decided to include all the women who met the inclusion criteria and who wanted to participate during the study period, even if the minimum number required for the sample was exceeded.

### 2.2. Information Source

An ad hoc questionnaire was used for data collection, which included questions on sociodemographic variables, obstetric information related to the neonatal state and feeding, and information on the treatment received. In addition, the discharge medical reports issued by the hospitals where the deliveries took place were required for collecting clinical information. Women could consult them for information.

This questionnaire was previously piloted to guarantee its readability and understandability. To recruit the participating women, we collaborated with midwives who provide care in health centers. When eligible women attended the consultations for the puerperal visit, these midwives informed the women about the study. If a woman decided to participate, they provided her with the link to the online questionnaire. The questionnaire was designed with easy and clear questions, with accessible language at all levels, without technicalities, and also with a simplified way to fill it out so that women only had to mark the answer option they wanted. The women were provided with the necessary instructions to be able to complete the questionnaire, and in addition, a telephone number and email address were available to the participants to clarify and resolve any issues.

### 2.3. Study Variables

The independent variables included sociodemographic variables such as maternal age, educational level, employment status, nationality, and monthly economic income; and obstetric variables such as parity, type of delivery, mode of labor onset, place of delivery, need for admission of the newborn, participation of the mother in a prenatal education program, presentation of a delivery plan and whether professionals had respected to this plan, early initiation of breastfeeding (in the first hour of the newborn’s life), performance of an episiotomy during childbirth, and the establishment of early skin-to-skin contact between the mother and the newborn. In addition, variables related to the support that the woman received from her partner during the process of pregnancy, childbirth, and puerperium were also collected.

The main dependent variable was the type of feeding of the newborn at the time of hospital discharge after delivery (exclusive/non-exclusive).

### 2.4. Statistical Analysis

First, descriptive statistics were carried out using absolute and relative frequencies for the qualitative variables. For quantitative variables, the mean with standard deviation (SD) were used. Next, a bivariate analysis was carried out between the different independent variables and the type of breastfeeding (exclusive/non-exclusive), using binary logistic regression and estimating odds ratios (OR) with their respective 95% confidence intervals. Subsequently, a multivariate analysis was performed using binary logistic regression to control for confounding bias. In the initial model, all the variables that had presented statistical significance in the bivariate analysis or that were identified in the previous literature as factors related to the type of feeding were included. The backward stepwise method was chosen to determine the final model, thus obtaining the adjusted OR (aOR) with its 95% confidence interval. Finally, the area under the ROC (receiver operating characteristic) curve was calculated to determine the predictive capacity of the final model. All analyses were performed with SPSS.

### 2.5. Ethical Considerations

The study received a favorable opinion from the Research Ethics Committee of the province of Jaen, reference number TD-VCDEPP-2019/1417-N-19. Before starting the questionnaire, the women had to read an information sheet about the study and its objectives and check a box in which they confirmed their consent to participate in it; that is, they signed a digital informed consent specifically elaborated for this study its manner of collecting the information.

## 3. Results

A total of 1200 women participated. Of these, 50.5% (606) were under 35 years of age, 65.4% (785) were primiparous, and 92.5% (1112) experienced a wanted pregnancy. Regarding the type of delivery, 58.5% (703) underwent a normal vaginal delivery, while 23.5% (283) underwent a cesarean section. In addition, 26.1% (313) underwent an episiotomy, while 72% (864) used epidural analgesia during labor. Early skin-to-skin contact after delivery occurred in 78.7% (945). Early breastfeeding was initiated in 75% (901). Regarding the type of breastfeeding after hospital discharge, in 77.8% (933) of the cases, the baby was fed by exclusive breastfeeding and in 18.3% (220) by mixed feeding, while 3.9% (47) chose to use formula. The mean length of hospital stay was 1.6 days (SD = 0.96) for women exclusively breastfeeding, 1.8 days (SD = 0.89) for women who used mixed feeding, and 2.10 days (SD = 0.91) for women who used formula feeding (Table 1).

Table 2 also shows the bivariate analysis between the different sociodemographic variables, obstetric variables, clinical practices, and the degree of partner support with the maintenance of exclusive breastfeeding after hospital discharge after delivery. The variables that showed a statistically significant association with exclusive breastfeeding after hospital discharge were: parity, multiple pregnancies, birth plan adhered to, skin-to-skin contact, labor induction, early breastfeeding, neonatal admission to a unit of care, feeling respected by professionals, breastfeeding in previous children, and regional or epidural analgesia, among others.

**Figure 1:**
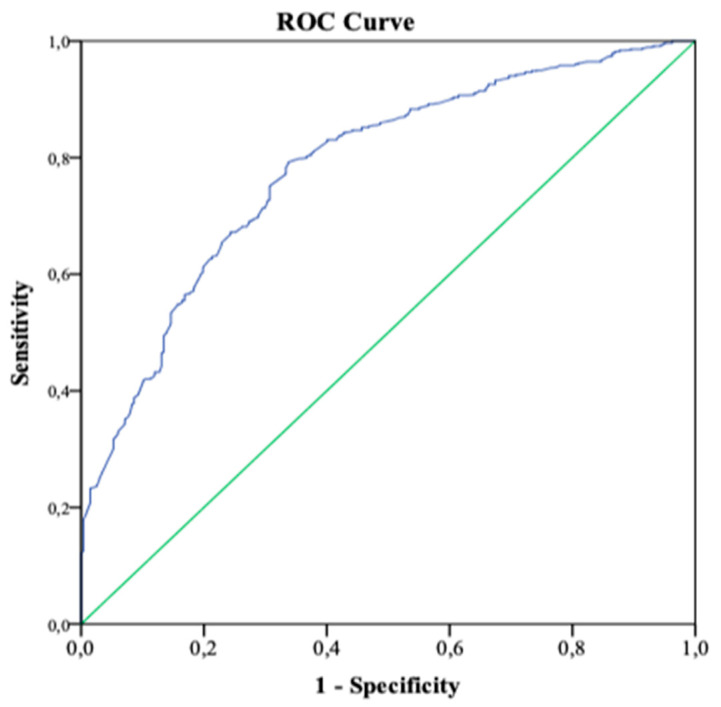
Predictive capacity of the model. The area under the ROC curve determines the predictive ability of the model in the study cohort. Sensitivity on the y-axis and 1-specificity on the x-axis.

However, after performing the multivariate analysis, factors that favored breastfeeding after hospital discharge were identified as having started breastfeeding early (aOR: 2.47; 95%CI: 1.77, 3.45), that the woman felt well supported by her partner during pregnancy, childbirth, and the puerperium (aOR: 2.54; 95%CI: 1.30, 5.00), as well as having a history of breastfeeding other children (aOR: 1.97; 95%CI: 1.40, 2.76). Factors that were associated with not maintaining exclusive breastfeeding after discharge included multiple or twin pregnancies (aOR: 0.31; 95% CI: 0.12, 0.83), induction of labor (aOR: 0.73; 95%CI 0.53, 0.99), admission of the newborn to the neonatal unit (NICU) (aOR: 0.31; 95%CI: 0.19, 0.52), use of regional or epidural analgesia during labor (aOR: 0.41; 95%CI: 0.27, 0.64), and premature birth (aOR: 0.38; 95%CI: 0.21, 0.69). The model’s predictive capacity (AUC-ROC) was 0.78 (95% CI: 0.75-0.81), as shown in Figure 1.

The predictive capacity (AUC-ROC) is 0.78 (95% CI: 0.75–0.81), as shown in Figure 1. Therefore, the predictive capacity is considered acceptable by the Swets criteria.

## 4. Discussion

More than 7 out of 10 women exclusively breastfed their babies in the present study. Among the factors that favored the maintenance of exclusive breastfeeding after discharge was its early initiation, that the woman felt very supported by her partner during pregnancy, childbirth, and the puerperium, and also previously breastfeeding other children. Multiple or twin pregnancies, labor induction, neonatal admission to intensive care, use of regional or epidural anesthesia during childbirth, and prematurity of the newborn appeared as factors associated with not exclusively breastfeeding after hospital discharge. 

In the present study, an online questionnaire was used for data collection. The online questionnaire has proven to be effective in collecting information associated with breastfeeding, [31] as long as it is approached rigorously, and it is also often the preferred method by participants [31,32]. Moreover, an anonymous and online questionnaire is a tool that facilitates honest responses from the participants, avoiding the self-reporting bias of “social-desirability” that may occur when the participant and the researcher maintain contact [33]. With the use of an online questionnaire, there may be a limitation in the participation of those women who do not have internet access or do not have the necessary skills to use this technology [34,35]. However, this possible bias was unlikely to have affected the data obtained, with only 53 women not responding despite being within the inclusion criteria. The questionnaire was adapted to the reference population, including for easy reading and understanding for any level of education, thus ruling out information bias. As the uptake occurred during the puerperal visit, memory bias can be almost completely ruled out as the information required for the questionnaire was recent. The study design also considered confounding bias, aiming to control it through the selection process and the analysis, and by adjusting the possible confounding variables. The objective of the study was to identify the factors associated with the maintenance of exclusive breastfeeding; although exclusive formula feeding is not the same as mixed feeding, these methods were grouped in the analysis of the variables.

The prevalence of exclusive breastfeeding after hospital discharge was 77.8%, a figure much higher than the rates previously described in different European countries [16,19]. This may be due to the absence of discrimination regarding the place of delivery, with both public and private hospitals participating in the baby-friendly hospital initiative, or “IHAN”, initiative. Of note, these initiatives are supported by various strategies that include providing mothers with information, as mothers with increased access to information about EBF are more likely to choose this method of infant feeding [36,37,38,39].

Early breastfeeding initiation is associated with EBF after hospital discharge, in line with that identified by other researchers who studied early initiation together with the early establishment of skin-to-skin contact [28,40,41]. Starting EBF during the first postpartum hour, regardless of the type of delivery, has been set as a fundamental practice to promote EBF [42]. Nonetheless, the support for EBF by professionals must continue even during the months after hospital discharge to maintain EBF rates [43].

Support from the partner during pregnancy, childbirth, and the puerperium appears to facilitate EBF, contrary to what was found by other authors. In a study conducted by Rempel et al. [44] in Canada in 2011 involving 34 couples, the authors found that when the parents were understanding and expressed the wish that mothers breastfeed their newborns longer, the duration of breastfeeding was shorter. At the same time, if the fathers were directly involved in breastfeeding, the duration of breastfeeding was also shorter. The researchers concluded that parental sensitivity and teamwork within the couple are the keys to prolonging EBF, as support needs to be responsive to the mother’s needs [44]. However, other authors suggest that this support from the partner is fundamental, which aligns with our findings. The intervention of health professionals in addressing the couple as a whole for EBF promotion plays a determining role [45]. Thus, considering our results, the partner and their support of EBF is decisive in the success of exclusive breastfeeding [44,45,46,47]. 

Having breastfed other children previously emerges as a facilitating factor for EBF after hospital discharge. Some studies analyzed the impact of exclusive breastfeeding in previous children as a factor promoting this method of feeding in subsequent children. Additionally, previous studies have considered whether the previous breastfeeding was carried out successfully, and the beneficial effects of antenatal interventions that were described in cases where there were problems with breastfeeding the first child [48]. This seems to indicate that the previous experience with breastfeeding affects the subsequent feeding method and may represent an opportunity for health professionals to detect previous problems in the obstetric history and anticipate them by improving the clinical care by providing targeted interventions that address the issues that led to non-successful breastfeeding in previous children. 

A multiple pregnancy appears to increase the difficulty in maintaining exclusive breastfeeding at hospital discharge, a finding that other authors have also previously reported [49,50,51,52]. Although few studies have been identified that analyze this factor and its relationship with EBF at hospital discharge in a developed country, researchers highlighted the training of professionals and mothers in breastfeeding, in addition to breast milk supplementation, as possible causes [51,52]. Mikami et al., in their prospective randomized trial carried out in Brazil with 128 women, also found similar results for women with multiple pregnancies [51].

The admission of the newborn to the neonatal ICU appears as another factor that negatively affects EBF after hospital discharge. In this regard, Vizzari et al. [23] obtained results similar to ours. Adapting the baby-friendly hospital initiative, increasing the training of health professionals who work in these units, and promoting a positive relationship between professionals and mothers are some of the solutions proposed by these researchers [23]. 

The present study’s results also indicate that professionals should include the partner in decision-making and conversations about EBF, as partner support can determine the success of this type of feeding after hospital discharge and in the long term [44,45]. Prematurity was also shown as a factor that makes EBF difficult. Different authors have reported similar results, and on many occasions, the challenges for EBF are increased by admission to intensive care of the newborn [53]. Although some supplementation to breast milk is often needed for the proper development of the premature baby, [21] this should not be a reason not to promote EBF, as it is crucial to do so early [54]. 

Labor induction also showed an association with non-maintenance of EBF after hospital discharge, a finding that coincides with those of other authors [24]. Zanardo et al., in an investigation carried out in Italy with 180 women, analyzed the rates of EBF at one month and three months postpartum in women who had undergone a labor induction process, showing a lower percentage of EBF than that in mothers who had not undergone labor induction [24].

If the mother used epidural analgesia during labor, she was less likely to be exclusively breastfeeding after hospital discharge. These results are consistent with those obtained by French et al., [25] although more research is needed. Within the cascade of events after analgesia, it may be the professional who attends the birth who should address the situation by anticipating and promoting exclusive breastfeeding together with early skin-to-skin contact between the mother and the newborn within the first hour postpartum in mothers who have an in situ epidural, thereby increasing the chances of success, as recommended by several authors [22,28].

These factors should be investigated further to determine the real impact on the continuity of EBF and thereby support the practice of health professionals. In particular, because health professionals have a role in promoting EBF and are also the vehicle that public administration policies use to promote this practice and thereby raise current EBF rates. The present study can provide a basis for follow-up during the first six months of a newborn’s life to identify the prevalence of EBF with the aim of implementing measures to improve EBF rates to levels in line with the current recommendations.

## 5. Conclusions

The early start of breastfeeding, feeling very supported by her partner during pregnancy, childbirth and the puerperium, or having breastfed previous children, all favor the maintenance of exclusive breastfeeding after hospital discharge. However, a multiple pregnancy, the induction of labor, admission to neonatal intensive care, epidural analgesia during birth, or prematurity of the newborn decrease the likelihood of exclusive breastfeeding after hospital discharge. A hospital following in the IHAN initiative’s footsteps seems to make a difference in maintaining exclusive breastfeeding beyond the hospital stay.

## Figures and Tables

**Table 1:** Sociodemographic and obstetric characteristics of the sample and the type of breastfeeding after hospital discharge.

Variable	Total *n* (%)	Type of Feeding (after Discharge)		
		Exclusive Breastfeeding	Mixed	Formula
Maternal age				
≤35 years	606 (50.5)	470 (77.6)	107 (17.7)	29 (4.8)
>35 years	594 (49.5)	463 (77.9)	113 (19.0)	18 (3.0)
Parity				
Primiparous	785 (65.4)	568 (72.4)	177 (22.5)	40 (5.1)
Multiparous	414 (34.6)	364 (87.9)	43 (10.4)	7 (1.7)
Education level				
Primary school	18 (1.5)	14 (77.8)	3 (16.7)	1 (5.6)
Secondary school	72 (6.0)	58 (80.6)	5 (6.9)	9 (12.5)
High school	258 (21.5)	182 (70.5)	63 (24.4)	13 (5.0)
University	852 (71.0)	933 (77.8)	220 (18.3)	47 (3.9)
Family monthly wage				
Less than 1000 euros	61 (5.3)	49 (8.3)	11 (18.0)	1 (1.6)
Between 1000 and 2000 euros	400 (33.3)	298 (74.5)	83 (20.8)	19 (4.8)
Between 2000 and 3000 euros	399 (33.2)	307 (76.9)	73 (18.3)	19 (4.8)
Between 3000 and 4000 euros	231 (19.2)	191 (82.7)	35 (15.2)	5 (2.2)
More than 4000 euros	109 (9.0)	88 (80.7)	18 (16.5)	3 (2.8)
Planned pregnancy				
No	88 (7.5)	71 (80.7)	15 (17.0)	2 (2.3)
Yes	1112 (92.5)	862 (77.5)	205 (18.4)	45 (4.0)
Maternal antenatal classes				
No	240 (20.0)	194 (80.8)	35 (14.6)	11 (4.6)
Yes (less than 5 classes)	171 (14.3)	127 (74.3)	37 (21.6)	7 (4.1)
Yes (more than 5 classes)	789 (65.7)	612 (77.6)	148 (18.8)	29 (3.7)
Twin pregnancy				
No	1177 (98.0)	924 (78.5)	207 (17.6)	46 (3.9)
Yes	23 (2.0)	9 (39.1)	13 (56.5)	1 (4.3)
Birth plan				
No	637 (53.0)	491 (77.1)	124 (19.5)	22 (3.5)
Yes, but not respected	137 (11.4)	85 (62.0)	43 (31.4)	9 (6.6)
Yes, and was respected	426 (35.6)	357 (83.8)	53 (12.4)	16 (3.8)
Skin-to-skin contact				
No	255 (11.3)	156 (61.2)	83 (32.5)	16 (6.3)
Yes	945 (78.7)	777 (82.2)	137 (14.5)	31 (3.3)
Type of conception				
Spontaneous	1063 (88.5)	839 (78.9)	183 (17.2)	41 (3.9)
Artificial insemination	23 (2.0)	18 (78.3)	5 (21.7)	0 (0.0)
IVF	114 (9.5)	76 (66.7)	32 (28.1)	6 (5.3)
Induction of labor				
No	709 (59.0)	584 (82.4)	103 (14.5)	22 (3.1)
Yes	491 (41.0)	349 (71.1)	117 (23.8)	25 (5.1)
Episiotomy				
No	887 (73.9)	700 (78.9)	154 (17.4)	33 (3.7)
Yes	313 (26.1)	233 (74.4)	66 (21.1)	44 (4.5)
Perineal tear				
No	724 (60.3)	542 (74.9)	149 (20.6)	33 (4.6)
Mild	434 (36.1)	355 (81.8)	69 (15.9)	10 (2.3)
Severe	42 (3.6)	36 (85.7)	2 (4.8)	4 (9.5)
Initiation of early BF				
No	299 (25.0)	169 (56.5)	95 (31.8)	35 (11.7)
Yes	901 (75.0)	764 (84.8)	125 (13.9)	12 (1.3)
Admission of the newborn to a care unit				
No	1038 (86.5)	847 (81.6)	156 (15.0)	35 (3.4)
Intermediate care	78 (6.5)	45 (57.7)	26 (33.3)	7 (9.0)
NICU	84 (7.5)	41 (48.8)	38 (45.2)	5 (6.0)
Feeling like the protagonist during pregnancy, childbirth, and puerperium				
No	110 (9.1)	63 (57.3)	35 (31.8)	12 (10.9)
A little	126 (10.5)	86 (68.3)	35 (27.8)	5 (4.0)
Somewhat	123 (10.4)	88 (71.5)	27 (22.0)	8 (6.5)
Considerably	316 (26.3)	244 (77.2)	62 (19.6)	10 (3.2)
A lot	525 (43.7)	452 (86.1)	61 (11.6)	12 (2.3)
Partner support during pregnancy, childbirth, and puerperium				
None	28 (2.5)	20 (71.4)	8 (28.6)	0 (0.0)
A little	52 (4.3)	34 (65.4)	16 (30.8)	2 (3.8)
Some	82 (6.8)	60 (73.2)	16 (19.5)	6 (7.3)
Considerable	273 (22.7)	208 (76.2)	57 (20.9)	8 (2.9)
A lot	765 (63.7)	611 (79.9)	123 (16.1)	31 (4.1)
Healthcare professionals respectful during pregnancy, childbirth, and puerperium				
No	53 (4.6)	30 (56.6)	17 (32.1)	6 (11.3)
A little	83 (6.9)	61 (73.5)	18 (21.7)	4 (4.8)
Somewhat	190 (15.8)	125 (65.8)	56 (29.5)	9 (4.7)
Quite	458 (38.1)	359 (78.4)	81 (17.7)	18 (3.9)
Very	416 (34.6)	358 (86.1)	48 (11.5)	10 (2.4)
Previously BF other children				
No	645 (53.7)	453 (70.2)	155 (24.0)	37 (5.7)
Yes	555 (46.3)	480 (86.5)	65 (11.7)	10 (1.8)
Natural analgesia				
No	970 (80.8)	736 (75.9)	194 (20.0)	40 (4.1)
Yes	230 (19.2)	197 (85.7)	26 (11.3)	7 (3.0)
Regional analgesia (epidural)				
No	336 (28.0)	303 (90.2)	28 (8.3)	5 (1.5)
Yes	864 (72.0)	630 (72.9)	192 (22.2)	42 (4.9)
General anesthesia				
No	1158 (96.5)	909 (78.5)	206 (17.8)	43 (3.7)
Yes	42 (3.5)	24 (57.1)	14 (33.3)	4 (9.5)
Preterm newborn				
No	1125 (93.7)	895 (79.6)	190 (16.9)	40 (3.6)
Yes	75 (6.3)	38 (50.7)	30 (40.0)	7 (9.3)
Type of birth				
Normal vaginal delivery	703 (58.5)	588 (83.6)	96 (13.7)	19 (2.7)
Instrumental	214 (17.8)	160 (74.8)	46 (21.5)	8 (3.7)
Elective C/S	82 (7.0)	52 (63.4)	28 (34.1)	2 (2.4)
Emergency C/S	201 (16.7)	133 (66.2)	50 (24.9)	18 (9.0)

**Table 2:** Association between the different variables and the maintenance of exclusive breastfeeding after hospital discharge. Bivariate and multivariate analysis.

Variable		Exclusive Breastfeeding (EBF)		
	No	Yes	OR 95%CI	aOR 95%CI
Maternal age				
≤35 years	136 (22.4)	470 (77.6)	1 (ref.)	
>35 years	131 (22.1)	463 (77.9)	1.02 (0.78, 1.34)	
Parity				
Primiparous	217 (27.6)	568 (72.4)	1 (ref.)	
Multiparous	50 (12.1)	364 (87.9)	**2.78 (1.99, 3.89)**	
Education level				
Primary school	4 (22.4)	14 (77.8)	1 (ref.)	
Secondary school	14 (19.4)	58 (80.6)	1.18 (0.34, 4.15)	
High school	76 (29.5)	182 (70.5)	0.68 (0.22, 2.15)	
University	173 (20.3)	933 (77.8)	1.12 (0.37, 3.45)	
Family monthly wage				
Less than 1000 euros	12 (19.7)	49 (8.3)	1 (ref.)	1 (ref.)
Between 1000 and 2000 euros	102 (25.5)	298 (74.5)	0.72 (0.37, 1.40)	0.68 (0.32, 4.43)
Between 2000 and 3000 euros	92 (23.1)	307 (76.9)	0.82 (0.42, 1.60)	0.82 (0.39, 1.73)
Between 3000 and 4000 euros	40 (17.3)	191 (82.7)	1.17 (0.57, 2.40)	1.23 (0.56, 2.70)
More than 4000 euros	21 (19.3)	88 (80.7)	1.03 (0.47, 2.26)	1.30 (0.54, 3.14)
Planned pregnancy				
No	17 (19.3)	71 (80.7)	1 (ref.)	
Yes	250 (22.5)	862 (77.5)	0.83 (0.48, 1.43)	
Maternal antenatal classes				
No	46 (19.2)	194 (80.8)	1 (ref.)	
Yes (less than 5 classes)	44 (25.7)	127 (74.3)	0.68 (0.43, 1.10)	
Yes (more than 5 classes)	177 (22.4)	612 (77.6)	0.82 (0.57, 1.18)	
Twin pregnancy				
No	253 (21.5)	924 (78.5)	1 (ref.)	1 (ref.)
Yes	14 (60.9)	9 (39.1)	**0.18 (0.08, 0.41)**	**0.31 (0.12, 0.83)**
Birth plan				
No	146 (22.9)	491 (77.1)	1 (ref.)	
Yes, but not adhered to	52 (38.0)	85 (62.0)	0.49 (0.33, 0.72)	
Yes, and was adhered to	69 (16.2)	357 (83.8)	**1.54 (1.12, 2.11)**	
Skin-to-skin contact				
No	99 (38.8)	156 (61.2)	1 (ref.)	
Yes	168 (17.8)	777 (82.2)	**2.94 (2.17, 3.97)**	
Type of conception				
Spontaneous	224 (21.1)	839 (78.9)	1 (ref.)	
Artificial insemination	5 (21.7)	18 (78.3)	0.96 (0.35, 2.62)	
IVF	38 (33.3)	76 (66.7)	0.53 (0.35, 0.81)	
Induction of labor				
No	125 (17.6)	584 (82.4)	1 (ref.)	1 (ref.)
Yes	142 (28.9)	349 (71.1)	**0.53 (0.40, 0.69)**	**0.73 (0.53, 0.99)**
Episiotomy				
No	187 (21.1)	700 (78.9)	1 (ref.)	
Yes	80 (25.6)	233 (74.4)	0.78 (0.58, 1.05)	
Perineal tear				
No	182 (25.1)	542 (74.9)	1 (ref.)	
Mild	79 (18.2)	355 (81.8)	**1.51 (1.12, 2.03)**	
Severe	6 (14.3)	36 (85.7)	2.02 (0.84, 4.86)	
Early BF initiated				
No	130 (43.5)	169 (56.5)	1 (ref.)	1 (ref.)
Yes	137 (15.2)	764 (84.8)	**4.29 (3.20, 5.75)**	**2.47 (1.77, 3.45)**
Admission of the newborn to care unit				
No	191 (18.4)	847 (81.6)	1 (ref.)	1 (ref.)
Intermediate care	33 (42.3)	45 (57.7)	**0.31 (0.19, 0.50)**	0.80 (0.44, 1.47)
NICU	43 (51.2)	41 (48.8)	**0.22 (0.14, 0.34)**	**0.31 (0.19, 0.52)**
Feeling like the protagonist during pregnancy, childbirth and puerperium				
No	47 (42.7)	63 (57.3)	1 (ref.)	
A little	40 (31.7)	86 (68.3)	1.60 (0.94, 2.73)	
Somewhat	35 (28.5)	88 (71.5)	**1.88 (1.10, 3.23)**	
Considerably	72 (22.8)	244 (77.2)	**2.53 (1.60, 4.01)**	
A lot	73 (13.9)	452 (86.1)	**4.62 (2.94, 7.26)**	
Partner support during pregnancy, childbirth, and puerperium				
None	8 (28.6)	20 (71.4)	1 (ref.)	1 (ref.)
A little	18 (34.6)	34 (65.4)	0.76 (0.28, 2.05)	1.89 (0.58, 4.20)
Some	22 (26.8)	60 (73.2)	1.09 (0.42, 2.83)	1.15 (0.58, 2.28)
Considerable	65 (23.8)	208 (76.2)	1.28 (0.54, 3.04)	1.80 (0.95, 3.43)
A lot	154 (20.1)	611 (79.9)	1.59 (0.69, 3.67)	**2.54 (1.30, 5.00)**
Healthcare professionals respectful during pregnancy, childbirth, and puerperium				
No	23 (43.4)	30 (56.6)	1 (ref.)	
A little	22 (26.5)	61 (73.5)	**2.13 (1.03, 4.41)**	
Somewhat	65 (34.2)	125 (65.8)	1.47 (0.79, 2.74)	
Quite	99 (21.6)	359 (78.4)	**2.78 (1.55, 5.00)**	
Very	58 (13.9)	358 (86.1)	**4.73 (2.57, 8.71)**	
Previously BF other child				
No	192 (29.8)	453 (70.2)	1 (ref.)	1 (ref.)
Yes	75 (13.5)	480 (86.5)	**2.71 (2.02, 3.65)**	**1.97 (1.40, 2.76)**
Natural analgesia				
No	234 (24.1)	736 (75.9)	1 (ref.)	
Yes	33 (14.3)	197 (85.7)	**1.90 (1.28, 2.82)**	
Regional analgesia (epidural)				
No	33 (9.8)	303 (90.2)	1 (ref.)	1 (ref.)
Yes	234 (27.1)	630 (72.9)	**0.29 (0.20, 0.43)**	**0.41 (0.27, 0.64)**
General anesthesia				
No	249 (21.5)	909 (78.5)	1 (ref.)	
Yes	18 (42.9)	24 (57.1)	**0.37 (0.20, 0.68)**	
Preterm newborn				
No	230 (20.4)	895 (79.6)	1 (ref.)	1 (ref.)
Yes	37 (49.3)	38 (50.7)	**0.26 (0.16, 0.43)**	**0.38 (0.21, 0.69)**
Type of birth				
Normal vaginal delivery	115 (16.4)	588 (83.6)	1 (ref.)	
Instrumental	54 (25.2)	160 (74.8)	**0.58 (0.40, 0.84)**	
Elective C/S	30 (36.6)	52 (63.4)	**0.34 (0.21, 0.55)**	
Emergency C/S	68 (33.8)	133 (66.2)	**0.38 (0.27, 0.55)**	

Bold: Statistically significant differences. OR: Odds ratio. aOR: Odds ratio adjusted.

## Data Availability

The data presented in this study are available on request from the corresponding author.
